# Hypoxia stimulates migration of breast cancer cells via the PERK/ATF4/LAMP3-arm of the unfolded protein response

**DOI:** 10.1186/bcr3373

**Published:** 2013-01-07

**Authors:** Anika Nagelkerke, Johan Bussink, Hilda Mujcic, Bradly G Wouters, Steffi Lehmann, Fred CGJ Sweep, Paul N Span

**Affiliations:** 1 Department of Radiation Oncology, Radboud University Nijmegen Medical Centre, Geert Grooteplein 10, 6525 GA Nijmegen, The Netherlands; 2 Department of Laboratory Medicine, Radboud University Nijmegen Medical Centre, Geert Grooteplein 10, 6525 GA Nijmegen, The Netherlands; 3 Ontario Cancer Institute, Campbell Family Research Institute, University Health Network, Departments of Radiation Oncology and Medical Biophysics, University of Toronto, 610 University Ave., Toronto, ON M5G 2M9, Canada; 4 Maastricht Radiation Oncology (MaastRo) Lab, GROW-School for Oncology and Developmental Biology, University of Maastricht, Universiteitssingel 50/23, 6229 ER Maastricht, The Netherlands; 5 Department of Cell Biology, NCMLS, Radboud University Nijmegen Medical Centre, Geert Grooteplein 10, 6525 GA Nijmegen, The Netherlands

## Abstract

**Introduction:**

The hypoxia-inducible factor (HIF)-1 pathway can stimulate tumor cell migration and metastasis. Furthermore, hypoxic tumors are associated with a poor prognosis. Besides the HIF-1 pathway, the unfolded protein response (UPR) is also induced by hypoxic conditions. The PKR-like ER kinase (PERK)/activating transcription factor 4 (ATF4)-arm of the UPR induces expression of lysosomal-associated membrane protein 3 (LAMP3), a factor that has been linked to metastasis and poor prognosis in solid tumors. In this study the role of UPR-induced LAMP3 in hypoxia-mediated migration of breast cancer cells was examined.

**Methods:**

A number of *in vitro *metastasis models were used to study the migration and invasion of MDA-MB-231 breast cancer cells under hypoxic conditions. PERK, ATF4 and their downstream factor LAMP3 were knocked down to examine their role in cell migration. In addition, multicellular tumor spheroids were used to study the involvement of the tumor microenvironment in invasion.

**Results:**

Using transwell assays, migration of different breast cancer cell lines was assessed. A direct correlation was found between cell migration and baseline LAMP3 expression. Furthermore, moderate hypoxia (1% O_2_) was found to be optimal in stimulating migration of MDA-MB-231 cells. siRNA mediated knockdown of PERK, ATF4 and LAMP3 reduced migration of cells under these conditions. Using gap closure assays, similar results were found. In a three-dimensional invasion assay into collagen, LAMP3 knockdown cells showed a diminished capacity to invade compared to control cells when collectively grown in multicellular spheroids.

**Conclusions:**

Thus, the PERK/ATF4/LAMP3-arm of the UPR is an additional pathway mediating hypoxia-induced breast cancer cell migration.

## Introduction

Breast cancer mortality is caused foremost by the spread of cancer cells within the host in a process called metastasis [1]. Before tumor cells can metastasize, the tumor will need to invade, seek access to the lymphatic or vascular system and colonize the metastatic site [2,3]. Insights in this process will aid in the prevention of cancer metastasis and help improve prognosis.

An important characteristic of most solid tumors is the presence of hypoxic regions [4-6]. Absent or inadequate vasculature within the tumor causes disruption of the supply of blood and consequentially an impaired delivery of oxygen and nutrients and an impaired removal of carbon dioxide and waste products. Several studies found low oxygen tension in tumors to be an adverse prognostic marker in different tumor types [7-10]. In addition, endogenous hypoxia-related markers, such as carbonic anhydrase-IX, were also shown to negatively influence patient outcome in breast cancer [11,12]. Furthermore, hypoxic tumors were found to correlate with metastatic occurrences: patients with hypoxic primary tumors developed more metastases than patients with less hypoxic tumors [7,13-15]. Mechanistically, numerous factors have been identified that are induced by hypoxia and that can promote metastasis (reviewed in [16-20]). The common denominator of most, if not all, of these factors is that they are either directly or indirectly influenced by the action of the family of master transcription regulators during hypoxic conditions: the hypoxia-inducible factor (HIF)-family [18].

Recently, a separate pathway from the HIFs was described, which is able to regulate gene expression during hypoxia, namely the unfolded protein response or UPR [21-24]. Within this response three distinct arms have been classified: the PKR-like endoplasmic reticulum kinase (PERK)/activating transcription factor 4 (ATF4)-arm, the inositol-requiring protein 1 (IRE1)-arm and the activating transcription factor 6 (ATF6)-arm. These pathways are activated during endoplasmic reticulum stress conditions and enable cell survival by regulating apoptosis, angiogenesis and autophagy [22-25]. Thus far, the UPR has not been directly implicated in hypoxia-induced metastasis. However, recently lysosomal-associated membrane protein 3 (LAMP3, also known as DC-LAMP, TSC-403 or CD208) was identified as a factor induced by hypoxia as part of the PERK/ATF4 arm of the UPR [26,27]. In addition, we found that LAMP3 has prognostic relevance in breast cancer [28]. Two homologs of LAMP3, LAMP1 and LAMP2, have been associated with cancer metastasis previously [29,30]. LAMP3 itself was also found to be involved in metastasis: overexpression of LAMP3 in a cervical xenograft model showed an increased metastatic potential [31]. In what way LAMP3 is involved in breast cancer metastasis and which role hypoxia may have in this process is unknown. Therefore, we set out to determine whether the UPR can influence migration and invasion of breast cancer cells via LAMP3 under hypoxic conditions.

## Materials and methods

### Cell culture and hypoxic incubations

All cell lines used were obtained from LGC Promochem (Teddington, UK) and maintained in Dulbecco's modified Eagle's medium (DMEM) supplemented with 10% (vol/vol) fetal bovine serum (FBS), 20 mM Hepes, 1 × nonessential amino acid, 2 mM L-glutamine and 10 U/ml penicillin, 10 μg/ml streptomycin (all from PAA Laboratories, Cölbe, Germany) at 37°C with 5% CO_2_. Hypoxic conditions were induced with a H35 Hypoxystation (Don Whitley Scientific Ltd, Shipley, UK).

### Cell migration using modified Boyden Chambers

Membranes with 8 μm pores (Greiner Bio-one, Alphen a/d Rijn, The Netherlands) were used in a 24-wells format. A total of 40,000 cells, serum-starved overnight where indicated, were added to the upper compartment. The lower compartment was filled with standard cell culture medium. Cells were allowed to migrate for 24 hours, after which chambers were fixed for 10 minutes in cold 70% ethanol. Membranes were stained with 0.5% (w/v) crystal violet (Sigma-Aldrich, St. Louis, MO, USA) for 30 minutes and subsequently washed thoroughly with tap water. Cells that had not migrated to the lower compartment were removed with a cotton swab. Migrated cells were quantified by solubilizing bound crystal violet in 1% (wt/vol) sodium dodecyl sulfate (SDS, Sigma-Aldrich) for 1 hour at 37°C. Absorbance was measured at 595 nm.

### RNA isolation, cDNA synthesis and quantitative polymerase chain reactions (qPCR)

RNA was isolated with Norgen's total RNA purification kit (Norgen Biotek Corp., Thorold, Canada) and stored at -80°C until further processing. cDNA was synthesized using the iScript cDNA synthesis kit (Bio-Rad Laboratories Inc., Richmond, CA, USA) with 1 μg RNA as input. The following primers were used for the qPCR: PERK FW: 5'-CTGATTTTGAGCCAATTC-3' and RV: 5'- CCGGTACTCGCGTCGCTG-3', ATF4 FW: 5'-CCTTCACCTTCTTACAACCT-3' and RV: 5'-GTAGTCTGGCTTCCTATCTC-3', LAMP3 FW: 5'-TGAAAACAACCGATGTCCAA-3' and RV: 5'-TCAGACGAGCACTCATCCAC-3'.

qPCR was carried out on a CFX96 real-time PCR detection system (Bio-Rad) with SYBR Green (Applied Biosystems, Foster City, CA, USA). As a reference gene, hypoxanthine-guanine phosphoribosyltransferase (HPRT) in a pre-developed assay (Applied Biosystems) was used.

### Transient transfection

Cells were transfected transiently for PERK, ATF4 or LAMP3 using mission siRNAs (Sigma-Aldrich):

PERK (NM_004836), SASI_Hs01_00096845 and SASI_Hs01_00096846

ATF4 (NM_001675), SASI_Hs02_00332313 and SASI_Hs01_00175197

LAMP3 (NM_014398), SASI_Hs01_00214233 and SASI_Hs02_00345584

Transfections were performed using Saint-Red (Synvolux Therapeutics, Groningen, The Netherlands) according to the manufacturer's instructions.

### Generation of stable MDA-MB-231 shLamp3 pools

A U6 promoter-driven short hairpin RNA (shRNA) expression vector targeting LAMP3 and a non-targeting control vector (PLKO1_shLAMP3 (TRCN0000148784, number 842) and PLKO.1 control, respectively) were purchased from Sigma-Aldrich. Briefly, pseudotyped lentiviral particles were produced in HEK293FT cells using the ViraPower lentiviral expression system according to the manufacturer's instructions (Invitrogen, Carlsbad, CA, USA). MDA-MB-231 cells were infected at a low passage number after which a pool of transfected cells was derived by puromycin (4 μg/ml) selection for approximately 10 days.

### Colony-forming assays

For the colony-forming assays 500 transiently transfected cells were plated in T25 cell culture flasks (Greiner Bio-one) and allowed to adhere overnight. Cells were incubated under hypoxic conditions (1% O_2_) for 24 hours after which they were transferred to the normoxic incubator and given time to form colonies. Once colonies in the normoxic controls comprised of at least 50 cells, flasks were fixated in 70% ethanol for 10 minutes at 4°C and stained with 0.5% crystal violet for 30 minutes. Colonies of at least 50 cells were scored manually.

### Gap closure assays

Monolayer gap closure assays (formerly known as scratch assays) were conducted using silicone cell culture inserts (Ibidi, Martinsried, Germany) attached to culture plates. In short, 30,000 cells were seeded in the inserts and allowed to recover overnight to form a confluent monolayer. Inserts were removed with tweezers, after which cells were rinsed thrice with Hank's buffered saline solution (HBSS, PAA Laboratories) to remove detached cells. Culture medium was re-added and closure of the gap was followed for 24 hours using live imaging (see below). Gap closure was quantified using ImageJ (National Institutes of Health, Bethesda, MD, USA).

### Spheroid culture

Multicellular tumor spheroids were prepared from conventional monolayer cultures using an adapted liquid overlay technique. In short, V-shaped 96-wells plates (Greiner Bio-one) were coated with 0.5% (wt/vol) poly-HEMA (Sigma-Aldrich). A total of 1,000 cells in 100 μl of standard culture medium with 2.5% (vol/vol) Matrigel (BD Biosciences, San Jose, CA, USA) were added to each well, after which cells were spun down for 10 minutes at 1,000 × g. Within 24 hours spheroids were formed.

### Pimonidazole staining

After formation, MDA-MB-231 spheroids were incubated for 1 hour with 200 μM of pimonidazole (1-[(2-hydroxy-3-piperidinyl)propyl]-2-nitroimidazole hydrochloride, Natural Pharmacia International Inc., Burlington, MA, USA). Next, spheroids were fixed in 4% paraformaldehyde (Merck Chemicals, Darmstadt, Germany), and embedded in paraffin. Staining was performed on 5-μm sections as previously described [28], using the following antibodies: mouse-anti-pimonidazole (Natural Pharmacia International Inc.) diluted 1:800 and biotin-conjugated donkey anti-mouse IgG (715-066-150, Jackson ImmunoResearch Laboratories Inc., West Grove, PA, USA) diluted 1:200.

### Cell labeling with cell tracker

To label cells with CellTracker Green (Life Technologies, Carlsbad, CA, USA) or CellTracker Orange (Life Technologies), 0.15 × 10^6 ^cells/ml were seeded in a T75 cell culture flask (Greiner Bio-one). Cells were allowed to adhere, after which CellTracker was dissolved in DMSO and added at 10 μM to the cell culture medium. Flasks were incubated at 37°C for 45 minutes. Next, cell culture medium was replenished and cells were allowed to recover for 30 minutes, after which they were ready for further experimentation.

### Spheroid invasion assays in collagen

Twenty-four hours after formation, spheroids were embedded into collagen. Spheroids were collected in a 15 ml tube and allowed to descend to the bottom. Cell culture medium was renewed once. Next, the spheroids were combined with 4 mg/ml of rat-tail collagen type I (BD Biosciences), according to the manufacturer's instructions. The collagen was allowed to polymerize for 10 minutes at room temperature, after which the mixture was pipetted carefully into a 12-wells plate (Greiner Bio-one). The collagen disk was incubated at 37°C until fully solidified, after which standard cell culture medium was added. Spheroids were allowed to invade the collagen for several days, as indicated in the legends to the figures.

### F-actin staining

After invasion of spheroids into collagen, collagen disks were fixed in 4% paraformaldehyde for 30 minutes at room temperature. Next, disks were washed with PBS, after which they were incubated with Alexa 488-conjugated Phalloidin (1:100; Life Technologies) for 3 hours at room temperature. After washing once more with PBS, images were acquired.

### Live imaging and microscopy

Live imaging of cells was performed using the JULI (Just Unbelievable Live Imaging) system from PAA. All other microscopic images were obtained using a Leica DM 6000 fluorescence microscope in combination with IPLab imaging software (Scanalytics Inc., Fairfax, VA, USA).

### Data analysis and statistics

Unless stated otherwise, all data are presented as mean values ± standard deviation. Statistical analysis was carried out using Student's *t *tests or one-way analysis of variance (ANOVA) with Tukey's post hoc test where appropriate unless stated otherwise. Two-sided *P *values < 0.05 were considered statistically significant. Asterisks indicate statistical significance: *** is *P *< 0.001, ** is *P *< 0.01 and * is *P *< 0.05.

## Results

### Breast cancer cell migration in transwell assays

**Figure 1:**
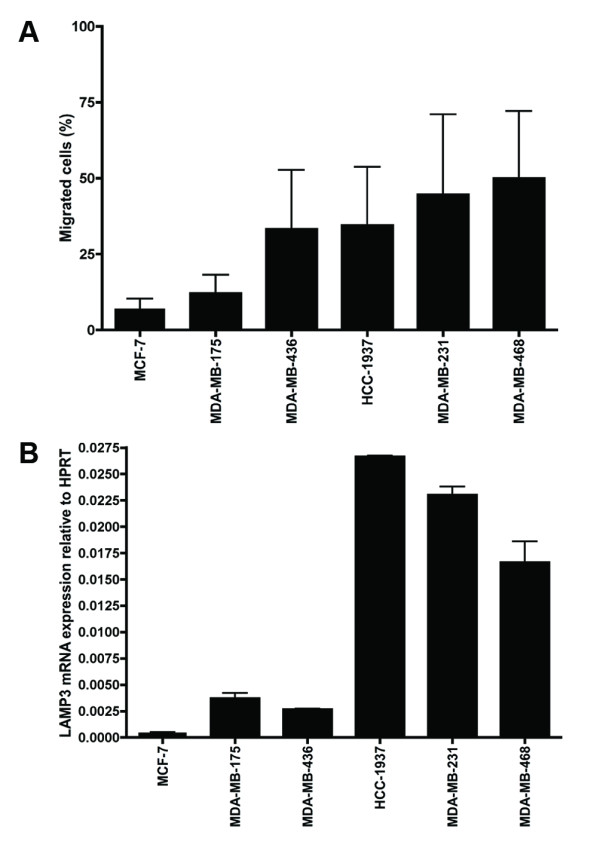
**Migration of breast cancer cells correlates with LAMP3 mRNA expression**. **(A) **Migration of six different breast cancer cell lines under normoxic conditions during 24 hours in a transwell assay. **(B) **mRNA expression of LAMP3 in the same six cell lines. Results are from two representative experiments with three replicates each. LAMP3, lysosomal-associated membrane protein 3.

To study the role of the UPR in the process of breast cancer cell migration, a number of models for *in vitro *metastasis were used. First, cell migration was studied in a series of six breast cancer cell lines under normoxic conditions using a transwell assay. In this assay cells have to actively migrate through a porous membrane. Cells were serum-deprived overnight and added to the upper compartment of modified Boyden chambers. In the lower compartment serum was added as a chemoattractant. As shown in Figure 1A, vast differences were found in the capability of cells to migrate to the lower compartment. The largest percentage of migrated cells was found in MDA-MB-231 and MDA-MB-468 cells, both triple-negative cell lines (that is, lacking expression of the estrogen receptor, progesterone receptor and human epidermal growth factor receptor 2). In contrast, MCF-7 and MDA-MB-175 cells demonstrated very little migration. A direct comparison between the potential to migrate and the expression of LAMP3 on the mRNA level revealed a moderate correlation in this small group of cell lines (see Figure 1B). All subsequent experiments were performed with the MDA-MB-231 cells, a commonly used breast cancer cell line to study cell migration.

### Importance of serum deprivation on migration of MDA-MB-231 cells in a transwell assay

**Figure 2:**
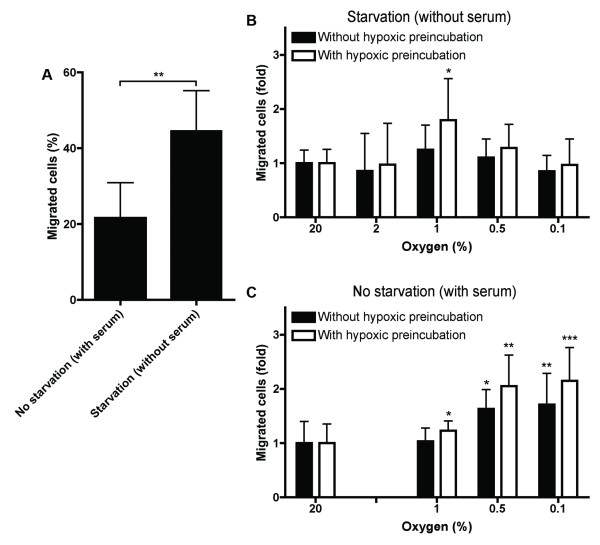
**Serum starvation and hypoxia increase cell migration in transwell assays**. **(A) **Effect of the addition of serum on the migration of MDA-MB-231 cells in a transwell assay during 24 hours. Effect of exposure to hypoxia on migration of MDA-MB-231 cells in a transwell assay under **(B) **serum-depleted or **(C) **serum-supplemented conditions, where cells were seeded in the transwell assays and then put in the hypoxic chamber for 24 hours (black bars) or cells received a 24 hour preincubation under the hypoxic conditions indicated on the x-axis and seeded in the transwell assays under hypoxia (white bars). Results are from two representative experiments with three replicates each. Asterisks indicate statistical significance of knockdown compared to the corresponding negative control.

The standard protocol for a transwell assay includes serum deprivation of the cells before the assay. This enables the addition of serum as a chemoattractant to the lower compartment. However, starvation induces the UPR profoundly and could therefore influence cell migration, especially under hypoxic conditions. In the present study, the chemoattractant function of serum was confirmed: overnight starvation of cells led to an increase in migration compared to cells that were not starved and thus allowed to migrate in complete medium (see Figure 2A). Next, this assay was used to establish the optimal percentage of oxygen to increase the migration of MDA-MB-231 breast cancer cells, in both serum-depleted and serum-supplemented conditions. When cells were serum-deprived overnight, cells migrated optimally at 1% O_2 _(see Figure 2B). Cell migration decreased at lower or higher oxygen concentrations. Subsequently, the assay was performed without starvation. The availability of serum influenced the optimal oxygen percentage profoundly. Figure 2C shows that in the presence of serum cells favored lower oxygen percentages for optimal cell migration (0.1% O_2_).

### Effect of knockdown of PERK, ATF4 and LAMP3 on cell migration in transwell assay during hypoxia

**Figure 3:**
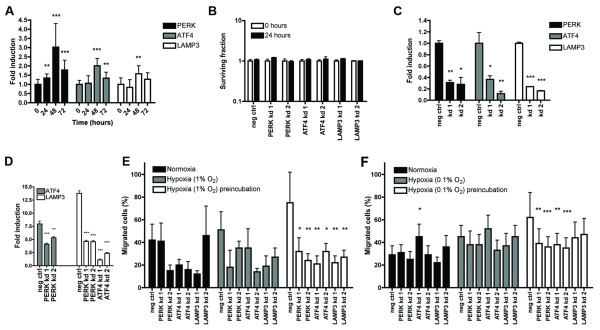
**Knockdown of PERK, ATF4 and LAMP3 reduces cell migration under hypoxic conditions in transwell assays**. **(A) **mRNA expression of PERK, ATF4 and LAMP3 after exposure of MDA-MB-231 cells to 1% O_2 _for the time indicated on the x-axis. **(B) **Clonogenic survival of MDA-MB-231 cells transiently transfected with siRNAs directed against PERK, ATF4 or LAMP3 after exposure to 1% O_2 _for a 24-hour period. **(C) **mRNA expression of PERK, ATF4 and LAMP3 in MDA-MB-231 cells after transient transfection with siRNAs directed against the corresponding genes. **(D) **mRNA expression of ATF4 and LAMP3 in MDA-MB-231 cells, transfected with siRNAs directed against PERK and ATF4, after serum starvation for 24 hours. **(E) **Effect of hypoxia (1% O_2_) in combination with transient knockdown of PERK, ATF4 or LAMP3 in MDA-MB-231 cells on cell migration in a transwell assay under serum-starved conditions. **(F) **Effect of hypoxia (0.1% O_2_) in combination with transient knockdown of PERK, ATF4 or LAMP3 in MDA-MB-231 cells on cell migration in a transwell assay under serum-supplemented conditions. Preincubated cells were exposed to hypoxic conditions 16 hours prior to the start of the assay. Assays were carried out under normoxic or hypoxic conditions during 24 hours. The preincubated assays were also performed under hypoxia. Results are from two representative experiments with three replicates each. Asterisks indicate statistical significance of knockdown compared to the corresponding negative control. ATF4, activating transcription factor 4; LAMP3, lysosomal-associated membrane protein 3; PERK, PKR-like endoplasmic reticulum kinase; siRNA, small interfering RNA.

Next, the effect of knockdown of PERK, ATF4 and LAMP3 on cell migration during hypoxia using the transwell assay was studied. First, we examined whether PERK, ATF4 and LAMP3 expression was induced by 1% O_2 _(see Figure 3A). Expression of these UPR components showed a moderate induction. Next, to ensure that any effects of knockdown on the migratory capacity during hypoxia were not due to reduced survival of the cells, a series of colony-forming assays was performed first. No significant effect of hypoxia (1% O_2_) on colony-forming ability of knockdown cells was found in the timeframe in which assays are performed (see Figure 3B). Figure 3C shows that transfection with the siRNAs used successfully diminished expression of the mRNA of PERK, ATF4 and LAMP3. In addition, knockdown of PERK and ATF4 reduced the expression of their downstream targets (that is ATF4 and LAMP3) after serum starvation (see Figure 3D). Subsequently, transwell assays were performed. First, cell migration through the porous membrane was studied at 1% O_2 _under serum-starved conditions. The most profound effect of knockdown on cell migration was induced when cells were preincubated under hypoxic conditions prior to the assay, which itself was also performed under hypoxia (see Figure 3E). Hypoxia without preincubation showed a diminished migration of cells for all knockdowns tested, but the effect was less profound than with the preincubated cells. Performing the assay in normoxic conditions led to a non-significant decrease in cell migration and only for some of the knockdowns tested. In addition, the cell migration after knockdown in a transwell assay at 0.1% O_2_, without serum starvation was also studied (see Figure 3F). Here, effects similar to the assay at 1% O_2 _were found, albeit less prominent.

### Migration of MDA-MB-231 breast cancer cells in a gap closure assay under hypoxic conditions

**Figure 4:**
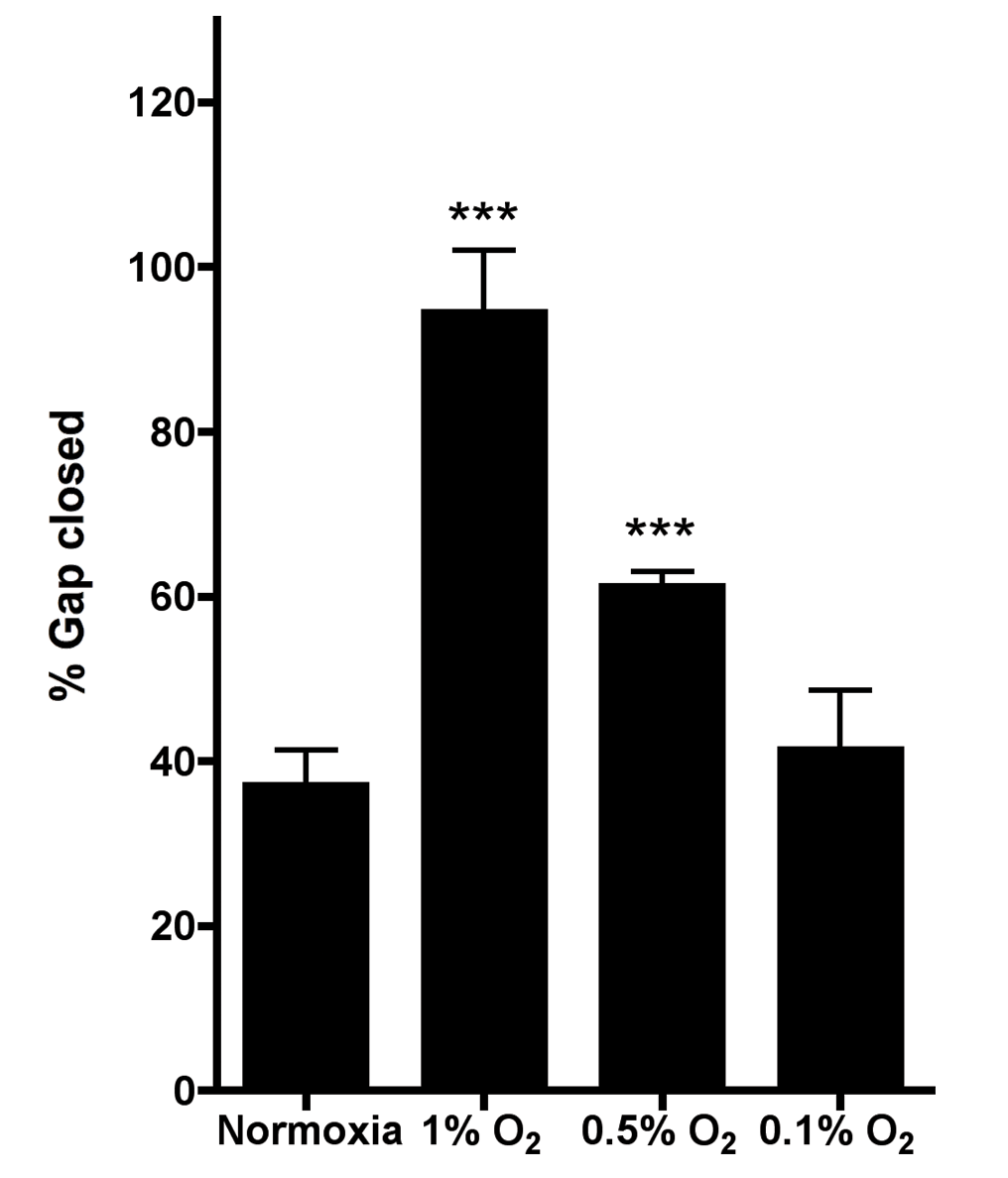
**Hypoxia has a profound effect on cell migration in a gap closure assay**. Shown is the percentage of the gap that is closed after 16 hours. Results are from two representative experiments with two replicates each. Asterisks indicate statistical significance compared to the normoxic control.

To confirm and extend the data from the transwell assay, similar experiments were performed using a gap closure assay, which does not require serum deprivation. First, the effect of exposure to hypoxia on the migration of MDA-MB-231 breast cancer cells was studied. Figure 4 shows the percentage of the gap that the cells filled in a 16-hour period. Compared to normoxic conditions, 1% O_2 _showed the largest percentage of gap closure. 0.5% O_2 _revealed a moderate but still significant effect. Exposure to even lower oxygen concentrations, that is 0.1% O_2_, did not reveal an increase in gap closure speed compared to normoxia.

### Effect of knockdown of PERK, ATF4 and LAMP3 on cell migration during hypoxia in a gap closure assay

**Figure 5:**
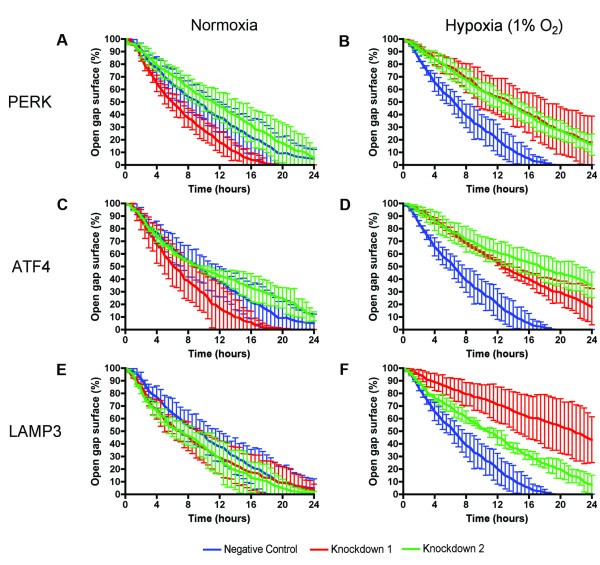
**Knockdown of PERK, ATF4 and LAMP3 reduces cell migration under hypoxic conditions in gap closure assays**. **(A-F) **Effect of hypoxia (1% O_2_) in combination with transient knockdown of PERK, ATF4 or LAMP3 in MDA-MB-231 cells on cell migration in a gap closure assay. Results are from two representative experiments. ATF4, activating transcription factor 4; LAMP3, lysosomal-associated membrane protein 3; PERK, PKR-like endoplasmic reticulum kinase.

As 1% O_2 _was found to be the optimal level of hypoxia for cells to migrate, subsequent gap closure experiments were performed at this oxygen level. Figure 5 shows the effect of knockdown of PERK, ATF4 and LAMP3 on migration of MDA-MB-231 cells. Under normoxic conditions no significant difference between cells transfected with a negative control and cells transfected with siRNA against PERK, ATF4 or LAMP3 could be demonstrated (see Figure 5A, C and 5E). In contrast, when the assay was performed under 1% O_2 _a considerable effect was observed (see Figure 5B, D and 5F). Knockdown of PERK, ATF4 or LAMP3 led to a substantial decrease in the speed at which cells close the created gap (see also Additional file 1).

### Importance of the tumor microenvironment for migration of MDA-MB-231 cells

**Figure 6:**
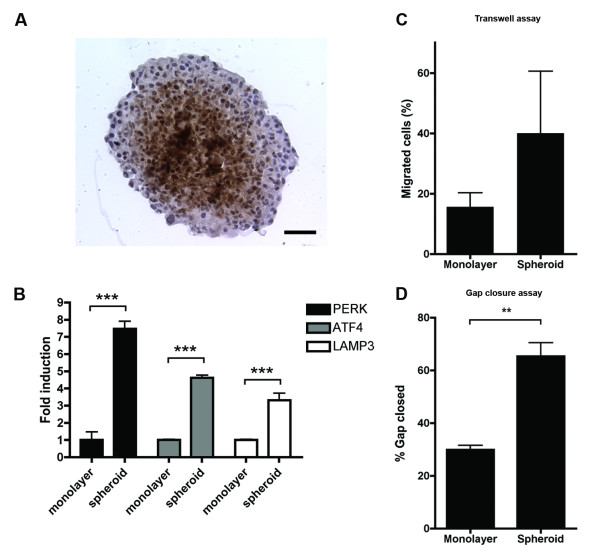
**Cells derived from tumor spheroids are more migratory than monolayer cells**. **(A) **MDA-MB-231 spheroid stained against the hypoxia marker pimonidazole. Bar is 100 μm, original magnification is 200 x. **(B) **mRNA expression of PERK, ATF4 and LAMP3 in MDA-MB-231 monolayers compared to cells grown as spheroids. Migration of monolayer cells compared to cell initially grown as a spheroid in a transwell assay **(C) **during 24 hours and a gap closure assay **(D) **during 16 hours in standard cell culture medium. Spheroids were disintegrated into a single-cell suspension prior to the start of the assay. Results are from two representative experiments with three replicates each. ATF4, activating transcription factor 4; LAMP3, lysosomal-associated membrane protein 3; PERK, PKR-like endoplasmic reticulum kinase.

All assays used so far required an artificial induction of hypoxic conditions. To study the effect of hypoxia on migration with endogenous hypoxia, multicellular tumor spheroids were employed. Beyond a certain diameter, tumor spheroids will develop a core consisting of hypoxic cells (see Figure 6A). Expression of PERK, ATF4 and LAMP3 was found to be higher in cells grown as a monolayer than cells grown in a spheroid (see Figure 6B). To directly compare the potential to migrate of a standard two-dimensional monolayer and three-dimensional spheroids both transwell and gap closure assays were performed. Directly before the start of the assays, spheroids were disintegrated by standard trypsinization into a single cell suspension, which was used as the input for the assays. In both assays the cells initially cultured as spheroids showed an increased capacity to migrate compared to the cells grown as a monolayer (see Figure 6C and 6D). Thus, once cells have experienced a simplified, transient tumor microenvironment, their migratory capacity increases.

### Effect of LAMP3 knockdown on collagen invasion of MDA-MB-231 spheroids

**Figure 7:**
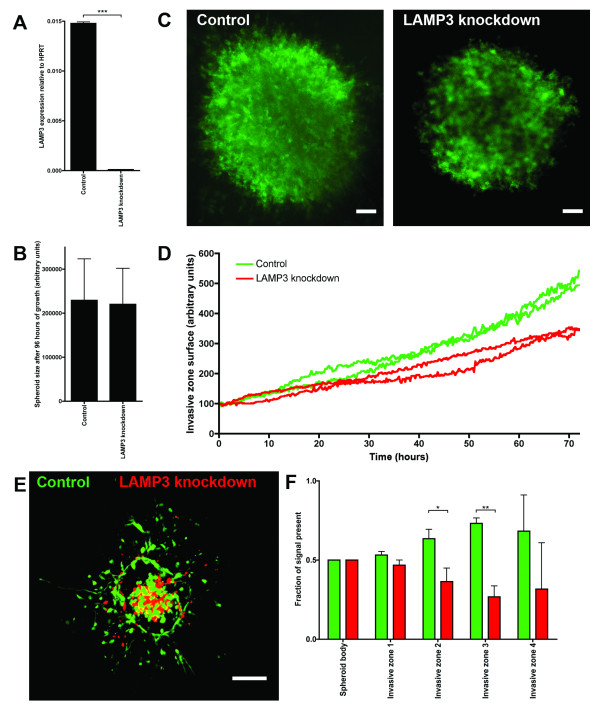
**Knockdown of LAMP3 reduces invasion of spheroids into collagen**. **(A) **Expression of LAMP3 mRNA in control cells versus LAMP3 knockdown cells. **(B) **Spheroid size of MDA-MB-231 control and LAMP3 knockdown spheroids after 4 days of growth. Results are from two independent experiments with 32 replicates each. **(C) **F-actin staining of MDA-MB-231 spheroids of control and LAMP3 knockdown cells, after 6 days of invasion in collagen. Bar is 100 μm, original magnification is 50 x. **(D) **Surface of collagen invaded by MDA-MB-231 control and LAMP3 knockdown spheroids during 72 hours. **(E) **Collagen invasion of a mixed MDA-MB-231 spheroid after 6 days. Control cells were labeled with CellTracker Green and LAMP3 knockdown cells were labeled with CellTracker Orange. Control and LAMP3 knockdown cells were mixed in a 1:1 ratio. Bar is 100 μm, original magnification is 100 x. **(F) **Quantification of E. The total amount of green and red signal was analyzed in five different zones: the spheroid body and four consecutive invasive zones, *n *= 5. LAMP3, lysosomal-associated membrane protein 3.

Next, the ability of spheroids generated from cells with a stable knockdown of LAMP3 to invade a collagen matrix was studied. Figure 7A shows that stable knockdown of LAMP3 attenuated the mRNA expression of LAMP3. After 4 days of growth, both control and knockdown spheroids were of similar size, indicating that there is no difference in proliferation between these cells when grown as a spheroid (see Figure 7B). MDA-MB-231 negative control spheroids were found to invade the collagen with string-like protrusions (see Additional file 2). Stable knockdown of LAMP3 reduced these invasive filaments to some extent (see Figure 7C and Additional file 2). The effect of LAMP3 knockdown on invasion of the spheroids into collagen was quantified by measuring the surface of collagen invaded by the spheroids. The surface of the invasive zone was found to be smaller in the knockdown spheroids (see Figure 7D).

The diminished invasion of LAMP3 knockdown spheroids was validated further by generating spheroids of a mixed origin. Control cells were labeled with CellTracker Green and LAMP3 knockdown cells were labeled with CellTracker Orange. Subsequently, mixed spheroids in a 1:1 ratio were generated. Invasion of these spheroids in collagen demonstrated the enhanced ability of the control cells to deeply invade the collagen compared to the LAMP3 knockdown cells (see Figure 7E). This effect was quantified by analyzing the green and red signal present within the spheroid body and the invasive zone (see Figure 7F). The invasive zone was divided into four circular zones with increasing distance from the spheroid body. Analysis of the signal present within each zone revealed that the green signal was more prevalent in the invasive zones than the red signal, indicating that the control cells invaded the collagen deeper than the LAMP3 knockdown cells.

## Discussion

In this study, evidence is provided that UPR-induced LAMP3 can influence hypoxia-mediated cell migration of breast cancer cells. We provide evidence that apart from the established involvement of the HIF-pathway in the induction of cancer cell spread, the UPR is a second manner in which hypoxia is implicated in breast cancer cell migration.

A characteristic of the UPR is its maximum induction under conditions of severe hypoxia (< 0.2% O_2_) or anoxia [32]. Indeed, LAMP3 as a UPR-induced factor was found to have its peak of induction under these conditions [26,28]. However, cell migration and invasion is often studied under conditions of more moderate hypoxia, around 1% oxygen [33-38], conditions which maximally induce HIF-1α expression [23]. The current study shows that knockdown of PERK, ATF4 or LAMP3 in combination with hypoxic exposure to 1% O_2 _led to a reduction in cell migration. If the UPR and its associated factors are maximally induced by anoxia, why are the largest effects observed at moderate levels of hypoxia? Several studies have shown that the UPR can indeed be induced by moderate hypoxia as well, but with different kinetics [21,39,40]. In this study assays were performed at 1% O_2 _as stronger hypoxic conditions did not lead to a stimulation of cell migration. Possibly, at severe hypoxic conditions, cells apply the UPR more for cell survival [25] than migration.

In the transwell assays an intriguing effect of the addition of serum on the induction of cell migration by hypoxia was observed. Serum-starved cells migrated most profoundly at 1% O_2_, whereas serum-supplemented cells migrated best at 0.1% O_2_. Serum dependency of cancer cell invasion has been observed before [41,42]. When MDA-MB-231 cells were serum depleted, no increase in invasion was found for a hypoxic incubation (1.5% O_2_) [42]. As addition of serum led to an increased invasion under hypoxia, it was suggested that serum might contain factors that increase invasion under hypoxic conditions [42]. In a different study, the effect of hepatocyte growth factor (HGF) on tumor invasion was examined in U2-OS and SiHa cells [43]. Mild hypoxia (3% O_2_) was found to increase invasion by amplifying HGF signaling, thereby sensitizing cells to HGF stimulation [43]. These and the current data indicate that the role of serum in cancer cell migration and invasion is more than just a chemoattractant and that growth factor signaling has a vast influence on the effects of hypoxia in migration and invasion assays. Trying to survive during hypoxia is of critical importance for cells. Depriving cells of serum may make survival even more difficult. Possibly, without serum and under severe hypoxic conditions (0.1% O_2_) cells are not migrating as fast as under moderate hypoxia (1% O_2_) because cell survival is more essential. When serum is present, the need for survival may become less critical even at 0.1% O_2_, causing the increase in cell migration.

The importance of the tumor microenvironment during cell migration was emphasized when migration assays were performed with cells grown as monolayer and cells initially grown as spheroids. The latter were found to be more migratory than the former. In other words, cells that experienced a simplified microenvironment prior to the assay were more migratory, despite the fact that spheroids had to be disintegrated back to a single-cell suspension. This behavior was also observed previously with murine breast cancer cell lines in transwell invasion assays [44]. As the spheroids used contain a central hypoxic core, the enhanced ability of spheroid cells to migrate or invade could be a consequence of hypoxia. This, however, remains to be established.

With the evidence that LAMP3 is involved in hypoxia-induced cell migration, it needs to be elucidated which mechanism LAMP3 uses to cause the actual spread of cancer cells. LAMP3 protein under physiological conditions is localized within the lysosomal membrane [45]. For LAMP1 and LAMP2 it has been previously established that their expression can relocalize to the plasma membrane in cancer cells [29]. Cell lines with a stronger metastatic capacity showed an enhanced expression of LAMP1 and LAMP2 on the cell membrane [29]. It is believed that LAMP surface expression provides a cancer cell with means to attach to selectins on endothelial cells and enhance their capacity to form metastases [30]. A similar mechanism could be responsible for the role of LAMP3 in hypoxia-induced cell migration. LAMP3 was found to have the ability to relocalize to the plasma membrane upon influenza A virus infections in HeLa cells [46]. Nevertheless, we have not been able to show LAMP3 expression on the cell surface in the MDA-MB-231 cells used, under either normoxic or hypoxic conditions. Immunohistochemically, in MDA-MB-231 cells we have only observed LAMP3 expression in the cytoplasm. An alternative explanation for the function of LAMP3 in cell migration is the link LAMP proteins have with autophagy [47]. Autophagy has been previously suggested as a possible mechanism responsible for increased survival and increased metastasis of cancer cells [48,49]. Analysis of the autophagy marker LC3B expression in a large subset of breast tumors revealed that it is associated with metastasis and a worse outcome [50]. However, Indelicato *et al*. found that chemically induced autophagy results in reduced invasion of MDA-MB-231 cells under both normoxic and hypoxic (1% O_2_) conditions, whereas LC3B silenced cells showed a decreased invasion during hypoxic conditions [37]. Thus, there is evidence that autophagy may function as a mechanism behind hypoxia-induced metastasis, but its precise role is far from clarified.

## Conclusions

In conclusion, this study provides evidence that the UPR with LAMP3 is involved in the process of hypoxia-induced cell migration. In addition, growth factor signaling via the serum component of cell culture medium is a factor of vital importance in the migration of cells under conditions of both moderate and severe hypoxia. Furthermore, the tumor microenvironment, experienced by cells when grown as multicellular spheroids is of significance in the process of cell migration and invasion. The PERK/ATF4/LAMP3 arm of the UPR might function as a new target for therapy combating hypoxia-induced metastasis in breast cancer.

## Abbreviations

ATF4: activating transcription factor 4; ATF6: activating transcription factor 6; DMEM: Dulbecco's modified Eagle's medium; FBS: fetal bovine serum; HBSS: Hank's buffered saline solution; HGF: hepatocyte growth factor; HIF: hypoxia-inducible factor; HPRT: hypoxanthine-guanine phosphoribosyltransferase; IRE1: inositol-requiring protein 1; LAMP3: lysosomal-associated membrane protein 3; PBS: phosphate-buffered saline pH 7.4; PERK: PKR-like endoplasmic reticulum kinase; qPCR: quantitative polymerase chain reaction; shRNA: short hairpin RNA; siRNA: small interfering RNA; UPR: unfolded protein response.

## Competing interests

The authors declare that they have no competing interests.

## Authors' contributions

Laboratory work and data analysis was performed by AN. The study was designed by AN, JB, FCGJS and PNS. SL created the stable LAMP3 knockdown cell lines. AN, JB, FCGJS and PNS wrote the manuscript. HM, BGW and SL critically revised the manuscript. All authors approved the final version of the manuscript.

## Supplementary Material

Additional file 1 **Effect of hypoxia (1% O_2_) in combination with transient knockdown of PERK, ATF4 or LAMP3 in MDA-MB-231 cells on cell migration in a gap closure assay**. Shown are time-lapse movies with 7 frames per second. Images were acquired during 24 hours at a 5-minute interval. Original magnification is 100 x. Click here for file

Additional file 2 **Invasion of MDA-MB-231 control and LAMP3 knockdown spheroids into a collagen matrix**. Shown are time-lapse movies with 7 frames per second. Images were acquired during 72 hours at a 15-minute interval. Original magnification is 200 x. Click here for file
